# A Crucial Role in Fertility for the Oyster Angiotensin-Converting Enzyme Orthologue *Cg*ACE

**DOI:** 10.1371/journal.pone.0027833

**Published:** 2011-12-09

**Authors:** Guillaume Riviere, Alexandre Fellous, Alban Franco, Benoit Bernay, Pascal Favrel

**Affiliations:** 1 UMR M100 Physiologie et Ecophysiologie des Mollusques Marins, Université de Caen Basse-Normandie - IFREMER, Caen, France; 2 Plateau de post-genomique ‘Proteogene’, IFR 146 ICORE, Caen, France; University of Cyprus, Cyprus

## Abstract

Angiotensin-converting enzyme (ACE) is a highly conserved metallopeptidase. In mammals, the somatic isoform governs blood pressure whereas the germinal isoform (tACE) is required for fertility. In Ecdysozoans, ACE-like enzymes are implicated in reproduction. Despite ACE orthologues being present from bacteria to humans, their function(s) remain(s) unknown in distant organisms such as Lophotrochozoans. *In silico* analysis of an oyster (*Crassostrea gigas)* EST library suggested the presence of an ACE orthologue in molluscs. Primer walking and 5′-RACE revealed that the 1.9 kb cDNA encodes *Cg*ACE, a 632 amino acid protein displaying a conserved single active site and a putative C-terminal transmembrane anchor, thus resembling human tACE, as supported by molecular modelling. FRET activity assays and Maldi-TOF spectrometry indicated that *Cg*ACE is a functional dipeptidyl-carboxypeptidase which is active on Angiotensin I and sensitive to ACE inhibitors and chloride ion concentration. Immunocytochemistry revealed that, as its human counterpart, recombinant *Cg*ACE is synthesised as a transmembrane enzyme. RT-qPCR, *in-situ* hybridization and immunohistochemistry shed light on a tissue, and development stage, specific expression pattern for *Cg*ACE, which is increased in the gonad during spermatogenesis. The use of ACE inhibitors *in vivo* indicates that the dipeptidase activity of *Cg*ACE is crucial for the oyster fertilization. Our study demonstrates that a transmembrane active ACE is present in the oyster *Crassostrea gigas*, and for the first time ascribes a functional role for ACE in Lophotrochozoans. Its biological function in reproduction is conserved from molluscs to humans, a finding of particular evolutionary interest especially since oysters represent the most important aquaculture resource worldwide.

## Introduction

Angiotensin-converting enzyme (ACE, dipeptidyl-peptidase A, kininase II, E.C. 3.4.15.1, DCP1) belongs to the M2-metalloprotease family and acts as a zinc-dependent dipeptidyl carboxypeptidase. In mammals, two isoforms are transcribed from two alternate promoters within the *ace-1* gene. The somatic isoform (sACE) is a crucial regulator of blood pressure especially since it generates the vasopressor angiotensin II (Ang II) from angiotensin I (Ang I). In contrast, the testicular (germinal) ACE (tACE) is required for fertility (reviews in [1], [2]) The *ace-1*gene originates from the duplication of an ancestral gene possessing a unique active site-coding region [3]. Consequently, sACE possesses two domains (the N-domain and C-domain, respectively) displaying the highly conserved gluzincin motif HExxH-23(24)-E. However, tACE possesses only one domain, corresponding to the C-terminal part of sACE. The N- and C- domains have distinct enzymatic specificities with respect to substrates, inhibitors [4] and chloride ion dependence [5]. Crystal structures of both the human N domain [6] and tACE [7], [8] have shown their active site to be a narrow catalytic channel connecting two large cavities within helical ellipsoids. Human ACE enzymes possess a C-terminal transmembrane domain and are membrane-anchored isoforms. Nevertheless, ACE can be released from the cell surface by a post-translational shedding [9]–[12], which can influence the biological role of ACE. An ACE homologue, ACE2, has also been characterized in humans [13], [14] and in mice [15].

A great diversity of catalytically active ACE-related enzymes has been found outside Vertebrates. In Ecdysozoans, ACE orthologues have been cloned in insects and crustaceans. Interestingly, only single active site proteins, that are synthesised as soluble [16]–[18] as well as transmembrane [19] proteins, were described. In the fruit fly *Drosophila melanogaster*, two homologous enzymes, AnCE [20] and Race [16] show about 40% amino-acid sequence identities with vertebrate ACEs. They also share similar enzymatic properties [21], yet their precise biochemical behaviour displays slight differences which are explained by structure/activity comparisons [22]. Moreover, active ACE orthologues in arthropods hydrolyse a broad range of substrates, and play a key role in reproduction [23]–[25], and development [26]–[28]. Interestingly, the ecdysozoan worm *C. elegans* possesses an ACE orthologue, ACN-1, with a role in development despite lacking an ACE-like proteolytic activity [29]. More distant in the evolution, a lophotrochozoan ACE protein was characterized in the leech which is related to the N-domain mammalian ACE [30]. However, its biological role is unknown despite molecular data suggesting a function in digestion. ACE is extremely conserved during animal evolution. Indeed, a functional soluble ACE orthologue is already present in the prokaryote *Xanthomonnas axonopodis pv citri* which is expressed in the bacterial periplasmic space [31]. Nevertheless, to our knowledge, all the attempts to address biological functions of ACE outside of Vertebrates and Ecdysozoa have been unsuccessful. Thus, these roles remain unknown despite such issues being fundamental in the understanding of both ACE evolution and physiology.

The pacific oyster *Crassostrea gigas* is a bivalve mollusc belonging to the Lophotrochozoa, a distant evolutionary group which remains extremely poorly described with respect to Ecdysozoa despite being its sister clade among protostomes. Furthermore, *C. gigas* is the most important aquaculture resource worldwide (FAO, 2003). Therefore, it is emerging as a model species, leading to the generation of a great amount of genomic expression data [32]. Interestingly, a partial cDNA sequence displaying similarity with ACE was identified within an EST library from gonads and early development stages of *C. gigas*. In order to gain more insight into ACE evolution and to investigate its putative activity and biological function in lophotrochozoans, we report the cloning and functional characterization of *Cg*ACE, the oyster ACE orthologue. The cDNA of *Cg*ACE was cloned and recombinant *Cg*ACE expressed in CHO cells. FRET assays and Maldi-TOF spectrometry were used to characterize *Cg*ACE activity. A homology-based model of *Cg*ACE was generated, and expression levels and localizations of the *Cg*ACE mRNA and protein were examined. *C. gigas* fecundations were also carried out in the presence of ACE inhibitors to address a putative biological function. To our knowledge, this study shows the first evidence of a biological role of ACE outside of vertebrates and ecdysozoa. *Cg*ACE is also the first molluscan ACE-like enzyme ever characterised.

## Materials and Methods

### Animals

Adult two-year old *Crassostrea gigas* specimens were purchased from an oyster farm (Blainville, Manche, France). Embryos, Larvae and spat were obtained at the IFREMER experimental hatchery (Argenton, France) [33]. Reproductive stage and sex were histologically determined as follows: stage 0 (sexual resting stage), male and female stage I (gonial multiplication stage), stage II (gametes maturation) and stage III (sexual maturity) [34].

### 5′-RACE PCR and primer walking

*In silico* analysis within the ‘Gigasdatabase’ oyster *Crassostrea gigas* EST database [32] revealed that three sequences produce significant homologies with the Angiotensin-converting enzyme (GenBank Accession numbers: CU989003, CU992640 and FP010921). These sequences were used to design oligonucleotides which were used downstream in 5′-RACE and primer walking strategies for the characterisation of the whole sequence of the ACE orthologue in *C. gigas*, named *Cg*ACE. Five prime rapid amplification of cDNA ends (5′-RACE) was performed on spat cDNA (Generacer kit, Invitrogen). Primer walking was conducted using the cDNA library plasmid as template, as previously described [30] (primers sequences and reaction conditions available upon request).

### Phylogenetic analysis

The whole protein sequence of the *Cg*ACE protein (Genbank accession number JN382542) was submitted to multiple sequence alignment with the ClustalW2 algorithm [35] (www.ebi.ac.uk) using the Gonnet matrix (parameters: gap open: 10; gap extension: 0,2; gap distance: 5; no end gap penalty; no iteration; numiter: 1; clustering method: neighbour-joining). The alignment file was used to generate a tree file with the neighbour-joining method using the Quicktree program v1.1 [36]. Based on this tree file, an unrooted tree diagram was plotted with the PHYLIP 3.67 Drawtree software [37] (http://mobyle.pasteur.fr).

### Molecular characterisation and expression

The full-length *Cg*ACE cDNA was amplified by PCR (primers: *Cg*ACEflS5 5′- CAC CTT AAC AAA CCA GAG AAG AGA AAG TCG AGG TG – 3′ and *Cg*ACEflAS1 5′- TAA ACA TGC CCG TTC CCA ATT TAT CCC TGC T -3′), subcloned into the pcDNA3.1 vector (Invitrogen) and sequenced. The obtained p*Cg*ACE plasmid was then co-transfected with pRL4-TK or pAcGFP (Promega) (9∶1) in CHO-K1 cells (ATCC n° CCL-61) using lipofectamine 2000 (Invitrogen). Cells were incubated for 24 to 48 h then assayed for ACE expression and activity (see below). For negative and positive controls, cells were respectively transfected using the empty vector (pcDNA3.1, mock), or a plasmid encoding the human wild-type sACE (pHswtACE, kindly provided by Annie Michaud, INSERM U883, College de France) in the same conditions. Renilla luciferase or GFP measurements were used to compensate for transfection efficiencies.

### RT-qPCR

Total mRNA was isolated from dissected organs (4 pools of 6 animals), spat after shell was removed, larvae or embryos as previously described [38]. Briefly, samples were extracted using Tri-Reagent (Sigma), then RNA were purified using affinity chromatography (Nucleospin RNA II kit, Macherey-Nagel). After digestion of genomic DNA with 1 U RQ1 DNAse (Promega) for 30 minutes to prevent genomic DNA contamination, 250 ng of total RNA were reverse-transcribed using 200 U of M-MLV RT (Promega) and 100 ng random hexamers. Resulting cDNAs were diluted and the equivalent amount of 5 ng of starting RNA was assayed for *Cg*ACE expression using actin (Genbank accession number: AF026063) and elongation-factor alpha (Genbank accession number: BAD15289) transcripts as reference genes. SYBR-green quantitative PCR was realised on an iCycler iQ© apparatus (Bio-Rad). Absolute Blue SYBRgreen Supermix (ThermoScientific) was used in 40 cycles (95°C/15 s, 60°C/15 s) reactions with the following primers: *Cg*ACE-F1 (5′-CAAGTGGAGATGGAGGGTGT-3′) and *Cg*ACE-R1 (5′-AACAGGAGGAGGTCACTTCCTT-3′); QaActin: (5′-CGTTGCCAATGGTGATG-3′) and QsActin (5′-GCCCTGGACTTCGAACAA-3′); or Qs-*Cg*-EF (5′-ACCACCCTGGTGAGATCAAG-3′) and Qa-*Cg*-EF (5′-ACGACGATCGCATTTCTCTT-3′) as sense and antisense primers respectively. Accurate amplification of the target amplicon was checked by performing a melting curve and an end-point agarose gel electrophoresis followed by ethidium bromide staining. A parallel amplification of reference genes was carried out to normalize the expression data of *Cg*ACE transcript. The relative level of *Cg*ACE expression was calculated for one copy of the reference gene by using the following formula: *N* = 2^(Ct Ref gene−Ct *Cg*ACE)^. Water was used instead of cDNA as a negative control for amplification, and DNAse-untreated cDNA was used to check for absence of genomic DNA contamination. All samples were analysed in triplicate to establish the mRNA expression profile of *Cg*ACE.

### Protein extraction and fractionation

Tissues or cell pellets were homogenized in ACE homogenization buffer (50 mmol.L^−1^ HEPES, 150 mmol.L^−1^ NaCl, 25 µmol.L^−1^ ZnSO_4_, 1 mmol.L^−1^ PMSF, 0,5% v/v CHAPS, pH = 6.5) and centrifuged (10000 g for 20 min. at 4°C). Cell culture media were harvested and concentrated when required on YM-100 and YM-10 centricon columns (Millipore). Supernatants containing total proteins and media were aliquoted and stored at −80°C. Cell proteins were fractionated as previously described [39]. Briefly, transfected cells (75 cm^2^ flasks) were scraped in homogenisation buffer (HB; 20 mmol.L^−1^ Tris–HCl pH = 7.2, 1 mmol.L^−1^ EDTA, 250 mmol.L^−1^ sucrose, 0,1 mmol.L^−1^ PMSF, 2 mmol.L^−1^ benzamidine), then centrifuged (1000 g, 5 min, 4°C). Supernatant was discarded and pellet was homogenised in 400 µL HB. The suspension was then ultracentrifuged at 100000 g for 1 h at 4°C. The supernatant was harvested (soluble fraction of cell proteins) and the pellet was washed with 1 ml HB, then resuspended in 400 µL HB (membrane fraction of cell proteins). Protein concentrations were determined using the Bradford method. For clarity, these protein extracts are termed in this study ‘recombinant *Cg*ACE’ and ‘recombinant HswtACE’.

### Immunological detection of *Cg*ACE

All immunological detections were performed using the sheep HKCE antiserum (kindly provided by Annie Michaud, INSERM U883, Collège de France, [31]) (dilutions: 1/5000 for immunocytochemistry, western blot and slot-blot; 1/1200 for immunohistochemistry). Anti-sheep IgG coupled to FITC (1/100, immunocytochemistry and slot-blot), alkaline phosphatase (1/300 immunohistochemistry) or horseradish peroxydase (1/200, western blot) were used as secondary antibodies. Western and slot-blots were carried out on 20 to 50 µg proteins, as previously described [40]. Slot-blot membranes were analysed for fluorescence signal using a ProXpress scanner (Perkin Elmer).

For immunocytochemistry, CHO-K1 cells were cultured on coverslips then transfected (see above). Cells were fixed in 4% paraformaldehyde for 10 minutes at 4°C then rinsed twice with ice-cold PBS. All incubations were performed at room temperature. Coverslips were mounted using DAPI containing mounting media and slides were observed under a confocal microscope (Olympus). For immunohistochemistry, oyster gonad samples (male and female stages I, II and III) were embedded in paraffin and serial 5 µm-sections deposited on poly-L-lysin slides. After paraffin removal and rehydration, slides were treated with hydrogen peroxide (0,3%) and saponin (0,05%) for antigen unmasking. The primary antibody was incubated overnight at 4°C. The secondary antibody was added for 1 hour at room temperature. Controls were realised on the same slides but omitting either the primary or the secondary antibody. Slides were histologically counter-stained using light green.

### ACE activity assays

Protein samples from total and/or fractionated extractions were assayed for ACE activity as follows:

#### FRET activity assays

*Cg*ACE hydrolysis of the human somatic ACE fluorogenic substrate Abz-FRK(Dnp)P-OH (Bachem) was determined in standard conditions as described previously [41]. Briefly, protein samples were incubated in activity buffer (140 mmol.L^−1^ NaCl, 5 mmol.L^−1^ KCl, 0,1 mmol.L^−1^ CaCl_2_, 0,63 mmol.L^−1^ MgSO_4_, 1 mmol.L^−1^ NaH_2_PO_4_, 6,1 mmol.L^−1^ glucose, 10 µmol.L^−1^ ZnSO_4_), in the presence or absence of ACE inhibitors (1.10^−6^ mol.L^−1^ Captopril (Sigma), Lisinopril or Fosinoprilat (kindly provided by Annie Michaud, INSERM U883, College de France)). FRK substrate (1.10^−5^ mol.L^−1^) was added and reactions were incubated for 1 to 2 hours at 37°C. Negative and positive controls were realised using pcDNA3.1- or pHswtACE-transfected cells extracts, respectively.

#### Influence of chloride ion concentration on HHL hydrolysis

A fluorescent assay was used to determine whether *Cg*ACE activity on the substrate hippuryl-histidyl-leucine (HHL) was sensitive to chloride ions concentration, using the procedure described previously [42]. Briefly, serum-free *Cg*ACE-transfected-cell culture medium were concentrated and desalted by diafiltration on cellulose columns (Millipore) and 7,5 µL of desalted concentrate were incubated in the presence of 5,7 mmol.L^−1^ HHL in potassium phosphate buffer containing increasing NaCl concentrations (from 0 to 1 mol.L^−1^) for 30 minutes at 37° C. Reactions were stopped by the addition of NaOH (final concentration: 0,25 mol.L^−1^). The presence of HL product at the end of the reaction was measured by the addition of α-phtaldialdehyde. Fluorescence development was stopped after 10 minutes by hydrochloride (final concentration: 0,3 mol.L^−1^). Fluorescence was measured at excitation 360 nm and emission 485 nm (Berthold Mithras 940 LE). Activity negative controls and fluorescence blanks were realized for each NaCl concentration. Specificity of the reaction was controlled by pre-incubations in the presence of ACE inhibitors (captopril, lisinopril and fosinoprilat at 1.10^−6^ mol.L^−1^ each for 15 minutes at room temperature), and by parallel assays using 7,5 and 5 µL of concentrated pcDNA3.1- or pHswtACE-transfected cell culture medium, respectively.

#### Mass spectrometry

*Cg*ACE was assayed for angiotensin I hydrolysis as described above, except that human angiotensin I (Sigma) was used instead of FRK as a substrate. The presence of angiotensin II at the end of the reaction was assayed using maldi/TOF-TOF mass spectrometry (MS) (AB Sciex 5800 proteomics analyzer, TOF/TOF ion optics and OptiBeam™ on-axis laser irradiation, 1000 Hz repetition rate). The system was calibrated immediately before analysis with a mixture of des-Arg-Bradykinin, Angiotensin I, Glu1-Fibrinopeptide B, ACTH (18–39), ACTH (7–38) (mass precision better than 5 ppm). Reaction solutions were mixed with CHCA matrix/50% ACN, 0.1% TFA. MS and MS/MS spectra were acquired in the positive reflector mode by summarizing 1000 single spectra (5×200, laser intensity 3000) in the mass range from 600 to 2000 Da and both m/z 1297 (Angiotensin I) and m/z 1046.7 (Angiotensin II), respectively.

### Molecular modelling of *Cg*ACE

A model of *Cg*ACE was generated using testis ACE (ProteinDataBank pdb code **1O8A**) as a template as previously described [31]. The *Cg*ACE sequence was submitted to the Esypred3D server (http://www.fundp.ac.be/sciences/biologie/urbm/bioinfo/esypred/) and alignments were obtained by combining, weighting and screening the results of several multiple alignment programs following the procedure described in [43]. Model comparisons were realized using the program DeepView [44]. Figures were generated with Pymol [45].

### *In situ* hybridization

A 856 pb fragment corresponding to the 3′-end of the *Cg*ACE cDNA, was amplified by PCR, subcloned into the pCRII vector (Invitrogen) and sequenced. The obtained plasmid was then linearized using NotI or EcoRI in separate reactions. The linearized product was resolved on agarose-gel electrophoresis stained with ethidium bromide and purified by affinity chromatography (Wizard SV gel purification, Promega). These products were used to generate sense and antisense *Cg*ACE digoxygenin-labelled riboprobes using SP6 and T7 RNA polymerases, respectively, and digoxygenin-dUTP (SP6/T7 RNA labelling kit, Roche). Paraffin-embedded tissues were cut (3 µm thick) and mounted on superfrost slides (VWR). Slides were deparaffined, rehydrated and hybridized overnight at 56°C. After extensive wash, digoxygenin-labelled hybridized probes were detected using an anti-digoxygenin alkaline-phosphatase antibody (Roche) and NBT-BCIP substrate (Sigma).

### Fecundation assays in the presence of ACE inhibitors

Broodstock *Crassostrea gigas* specimens were purchased from an oyster farm in Guernsey (Guernsey, GB) or obtained in the IFREMER experimental hatchery (Argenton, France). Gonads were scarified and gametes were filtered on a 100 µm mesh for the removal of large debris. For females, oocytes (oo) were harvested as the remaining fraction on a 30 µm mesh; for males, spermatozoa (spz) were harvested as the passing fraction on a 30 µm mesh. Spermatozoa were pre-incubated in filtered-sterile (0,22 µM) seawater (FSW) alone or in the presence of 10^−8^ to 10^−3^ mol.L^−1^ ACE inhibitors for 10 minutes at 25°C. Fertilizations were triggered by the addition of oocytes and were carried out in oxygenated FSW at 25°C (500 oo.L^−1^; ca. 100 spz/oo). Fertilization rates were determined as the number of at least two cell embryos within the total egg number after 2 hours.

### Statistical analysis

All the results are given as the mean +/− s.e.m. (standard error to the mean) of at least triplicate experiments. The results were analysed for statistical significance using two tailed Student's t test, one-way or two-way ANOVA followed by Bonferroni's post-hoc test. p<0.05 was considered significant. Data were analysed using the Graphpad Prism software version 5.0.

## Results

### Molecular characterisation of *Cg*ACE

**Figure 1:**
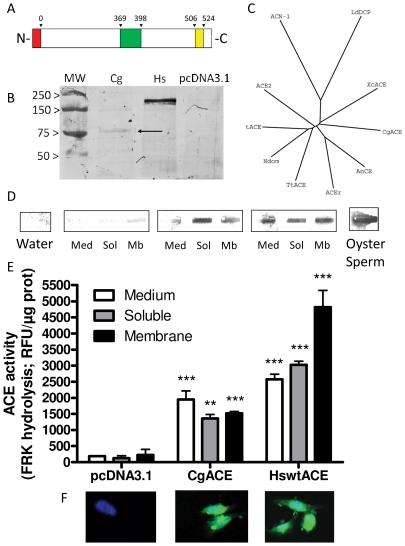
Molecular characterization of *Cg*ACE. A: schematic representation of the *Cg*ACE protein primary sequence. B: unrooted tree diagram showing the evolutionary relationship between human (tACE, Ndom, ACE2), fly (AnCE, ACEr), *C. elegans* (ACN-1), leech (*Tt*ACE), leishmania (*Ld*DCP), bacteria (*Xc*ACE) and oyster (*Cg*ACE) ACE orthologues (see Table 1). C: western blot; MW, molecular weight marker; Cg, recombinant *Cg*ACE; Hs, recombinant human wild-type sACE, pcDNA3.1, protein extract from mock-transfected cells. D: Slot blot. The subcellular origin of proteins analysed (Med, culture medium; Sol, soluble fraction of cell proteins; Mb, membrane fraction of cell proteins) is indicated. E: ACE activity in cell fractions. The rate of FRK fluorigenic substrate hydrolysis by recombinant *Cg*ACE and HswtACE and the origin of protein extracts are indicated. **: p<0.01, ***: p<0.001, two way ANOVA followed by Bonferroni's post hoc test, vs pcDNA3.1. F: immunocytochemistry of cells transfected with pcDNA3.1 (left), p*Cg*ACE (middle) or pHswtACE (right). Blue signal indicates nucleus (DAPI) and green signal indicates ACE detection (FITC) (see methods).

The cloning strategies allowed the characterization of a ∼2 kb cDNA exhibiting an in-frame 5′ stop codon, a 1896 base pairs coding sequence and a conserved polyadenylation signal. The aforementioned *Crassostrea gigas* Angiotensin-I converting enzyme cDNA sequence, named *Cg*ACE, was deposited in the Genbank database with the accession number JN382542. After signal peptide cleavage (A^19^/R^20^), the mature *Cg*ACE is predicted to be a 613 residues protein bearing a single conserved gluzincin motif HHEMGH(24)E starting at position 369, and a putative C-terminal transmembrane anchor (A^506^ to Y^524^) (Figure 1A and Supplementary Figure S1). Western blot of *Cg*ACE displays a single band slightly higher than 75 kDa, in line with the theoretical prediction of 74 kDa when considering putative post-translational modifications such as glycosylation (T^136^, T^350^, S^425^) (Figure 1B). Reminiscent of its human sACE counterpart, recombinant *Cg*ACE protein (Figure 1D) and ACE activity (see below) (Figure 1E) were detected not only within membrane protein fraction, but also within the soluble fraction and in the culture medium. Furthermore, recombinant *Cg*ACE and HswtACE exhibit a similar cell distribution (Figure 1F) when expressed in CHO-K1 cells.

### Phylogenetic position

*Cg*ACE exhibits significant similarity with other functional ACEs throughout the animal kingdom, but little if any with the inactive Caenorhabditis ACN-1 orthologue (Table 1). Concerning evolutionary position, *Cg*ACE clusters between the drosophila (AnCE and ACEr) and the bacterial orthologues, despite showing a greater similarity with the human and leech enzymes (Figure 1C). This result is obtained regardless of the score matrix used for sequence alignments (Gonnet or BLOSUM, respectively, data not shown).

**Table 1:** Similarity between ACE-like proteins throughout the animal kingdom.

	tACE	Ndom	ACE2	AnCE	ACEr	ACN-1	*Tt*ACE	*Xc*ACE	*Ld*DCP
tACE									
Ndom	51								
ACE2	39	39							
AnCE	43	39	35						
ACEr	38	36	34	49					
ACN-1	13	2	2	1	2				
*Tt*ACE	45	45	38	39	36	2			
*Xc*ACE	34	36	33	30	30	9	33		
*Ld*DCP	6	5	8	5	8	5	6	7	
***Cg*** **ACE**	**41**	**38**	**34**	**34**	**35**	**6**	**38**	**33**	**9**

The homology is given as a score representing the percentage of identity between proteins as given by the Gonnet matrix (see methods); tACE, human tACE (GI: 23238214); Ndom, human sACE N-domain (GI: 113045 residues 1–612); ACE2, human ACE2 (GI: 42543475); AnCE, *Drosophila melanogaster* ACE (GI: 10728771); ACEr, *Drosophila melanogaster* ACE-related (GI: 17137262); ACN-1, *Caenorhabditis elegans* non-peptidase ACE (GI: 71985293); *Tt*ACE, leech *Theromyzon tessulatum* ACE (GI: 45272589); *Xc*ACE, *Xanthomonas axonopodis pv.citri* ACE (GI: 21241971); *Ld*DCP, *Leishmania donovani* dicarboxypeptidase (GI: 56130986); *Cg*ACE: *Crassostrea gigas* ACE (Genbank accession number: JN382542).

### Biochemical characterisation of *Cg*ACE

**Figure 2:**
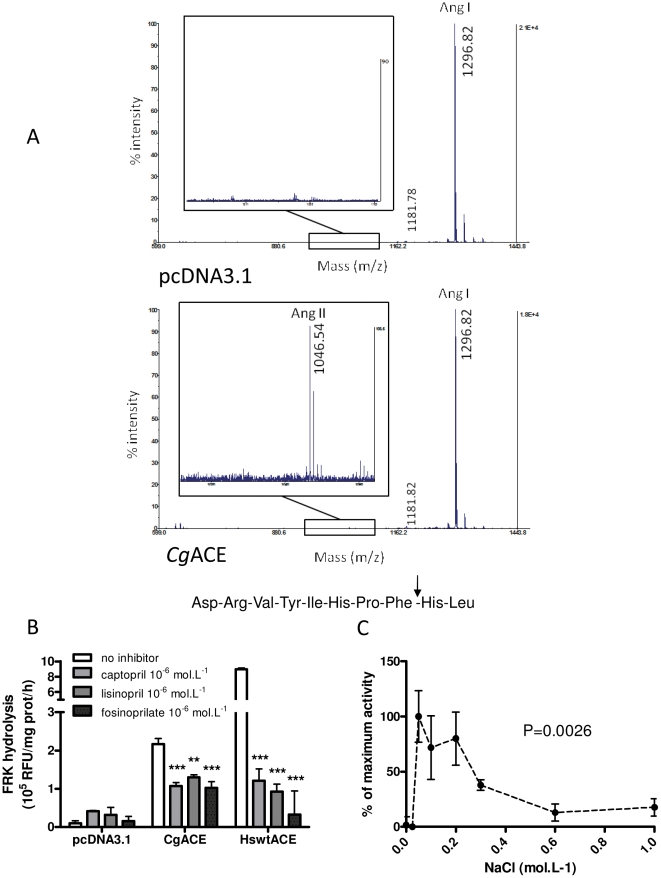
Biochemical characterization of *Cg*ACE. **A:** MS spectra of human Angiotensin I hydrolysates after incubation with protein extracts from pcDNA3.1-transfected cells (top) and recombinant *Cg*ACE (bottom). Peptide and peak masses (m/z), relative intensity (left Y axis) and signal intensity (right Y axis) are indicated. The 550–1500 and 1000–1100 m/z ranges are represented using different magnifications for clarity. AngI: angiotensin I, AngII: angiotensin II. The peptide sequence of angiotensin I is given, the arrowhead indicates the cleavage site leading to angiotensin II formation. **B:** Influence of 10^−6^ mol.L^−1^ ACE inhibitors on FRK hydrolysis by protein extracts from cells transfected with the vector backbone (pcDNA3.1), p*Cg*ACE or pHswtACE. The inhibitors used and their concentrations are indicated. **: p<0.01, ***: p<0.001, one way ANOVA followed by Dunnett's post hoc test, vs no inhibitor. **C**: influence of chloride ion concentration on HHL hydrolysis by *Cg*ACE. The p value for one way ANOVA of chloride concentration influence on *Cg*ACE activity is given.

Maldi-TOF mass spectrometry indicates that *Cg*ACE cleaves a C-terminal dipeptide from angiotensin I and therefore generates angiotensin II (Figure 2A). Indeed, the MS/MS data obtained from synthetic AngII on the one hand, and from the 1046 m/z peptide resulting from AngI hydrolysis by *Cg*ACE on the other hand, indicates that both peptides present the same sequence (DRVYIHPF) (Supplementary Figure S2). In addition, *Cg*ACE is able to hydrolyse a specific ACE substrate, i.e. Abz-FRK(Dnp)P-OH. In line with what was observed for AngI hydrolysis, this activity is significantly reduced in the presence of specific ACE inhibitors such as captopril, lisinopril and fosinoprilat (figure 2B). Nevertheless, in the reaction conditions used herein, these inhibitors are less potent for *Cg*ACE than for the human ACE. Indeed, micromolar concentrations decrease *Cg*ACE activity by ca. 40 to 50% instead of 90 to 95% in human tACE (Figure 2B). *Cg*ACE is also able to hydrolyze the HHL substrate. *Cg*ACE activity on HHL is sensitive to chloride ion concentration. Indeed, the enzyme displays maximum activity for concentrations around 0,05 mol.L^−1^ and exhibits only little activity (ca. ∼15% of maximum activity) for concentrations above 0,5 mol.L^−1^ (Figure 2C). In this regard, *Cg*ACE exhibits similar characteristics to the N-domain of human sACE when compared to the C-domain.

### Homology model of *Cg*ACE

**Figure 3:**
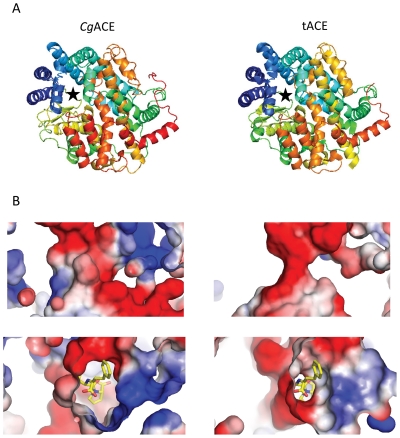
Molecular modelling of *Cg*ACE. Comparison between the *Cg*ACE model (left) and the human tACE structure (right). **A**: Ribbon representation of the overall conformation of the mature proteins. The active site is indicated (black asterisk). Spectrum colours indicate the position within the protein sequence from the N-terminal (blue) to the C-terminal (red) ends. **B**: Comparison of the active site between *Cg*ACE and tACE. ACE is represented as a surface with the positive charge indicated in blue and the negative in red. Lisinopril (in yellow) is shown to orientate the active site. Magnified views of the catalytic channel region are represented (top view with lysyl binding pocket down, upper panel; front view from the lysyl binding pocket, bottom panel).

As the crystal structures of the human ACE [6], [7], ACE2 [46] and *Drosophila* AnCE [47] all display the same fold, a structural model was generated to understand the interactions of residues in the active site. The pair-wise alignment of human tACE and oyster *Cg*ACE sequences revealed a 41% sequence identity that allowed us to confidently model the overall fold of *Cg*ACE based on the tACE structure. The homology modeling indicates that *Cg*ACE would closely resemble the human tACE in terms of overall conformation (Figure 3A). The primary structure of *Cg*ACE includes all the residues implied in zinc (H^369^, H^373^ and E^397^), lisinopril (H^339^, A^340^, E^370^, K^501^, H^503^, Y^510^ and Y^513^) and captopril (Q^226^, K^501^, Y^510^, H^339^, H^503^ and Y^513^) binding. Nevertheless, the structural model indicates that the catalytic channel of *Cg*ACE would be narrower than the human tACE active site (Figure 3B).

### *In vivo* Expression and activity of *Cg*ACE

**Figure 4:**
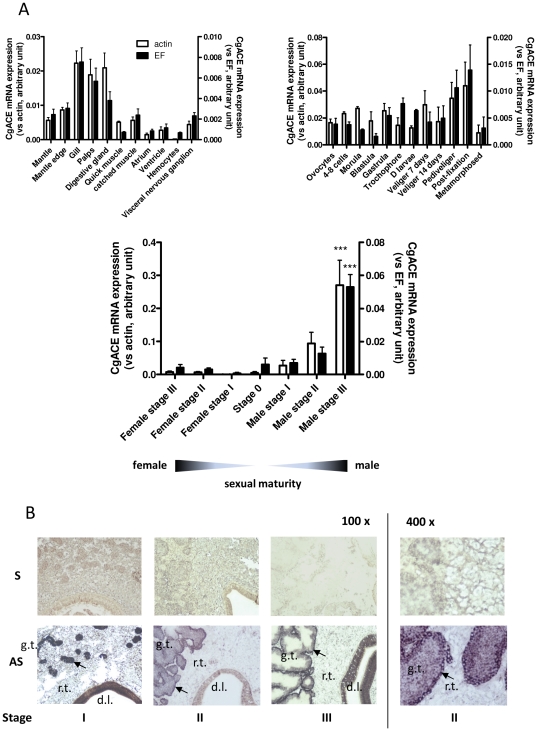
mRNA expression of *Cg*ACE. **A**: Relative *Cg*ACE mRNA expression levels in adult tissues (upper left panel), development stages (upper right) and gametogenesis (bottom). The expression level and reference genes are indicated. The bottom diagram represents the sexual maturation. ***: p<0.001, two-way ANOVA followed by Bonferroni's post hoc test. **B**: *Cg*ACE *in situ* hybridization in adult oysters. S, sense riboprobe; AS, antisense riboprobe. The magnification (100×, 400×) and the sexual maturation stage (I, II and III) are indicated; g.t., gonadic tubule; r.t. , reserve tissue ; d.l., digestive lumen. Arrowheads indicate typical signal localization.

**Figure 5:**
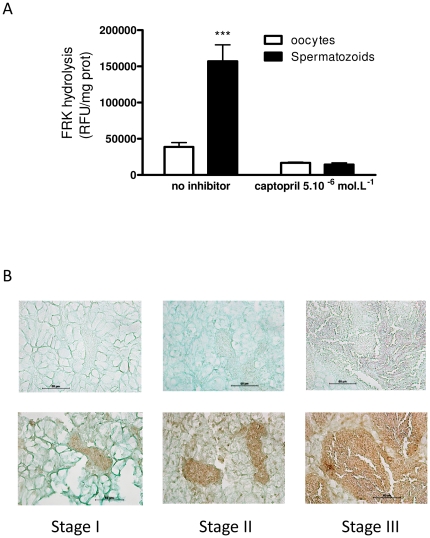
ACE activity in gametes and protein expression in gonads of *C. gigas.* **A**: FRK fluorigenic substrate hydrolysis by protein extracts from oyster gametes in the absence (no inhibitor) or the presence of 5.10^−5^ mol.L^−1^ captopril. **B**: Immunohistochemistry of *Cg*ACE in adult oysters. The corresponding control slides (top pictures, no primary antibody) are shown. The sexual maturation stage (I, II and III) is indicated.

The *Cg*ACE transcript shows little if any expression within adult tissues, except in the digestive tract (labial palps, digestive gland) and the gills. Similar expression levels are detected within the development stages of the oyster. In contrast, RT-qPCR reveals that the *Cg*ACE messenger RNA displays a dramatic increase in the gonadal area along with the spermatogenesis and spermiogenesis, but no significant change during female gametogenesis (Figure 4A). *In situ* hybridization indicates that the *Cg*ACE mRNA is highly expressed in the germinal compartment of the gonad; i.e. mostly spermatogonia, spermatocytes I and spermatocytes II (Figure 4B). No specific signal could be detected within female gonads (data not shown). Consistently, a great specific ACE activity was found in sperm protein extracts. Interestingly, little specific ACE activity could be detected within stripped oocyte protein extracts (Figure 5A). The *Cg*ACE protein was present within the gonadic tubules of male oysters (Figure 5B), while no specific signal could be detected within the female gonadal area (data not shown).

### Biological function of *Cg*ACE

**Figure 6:**
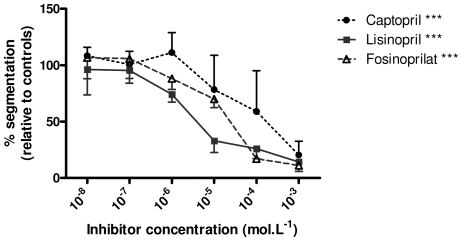
Influence of ACE inhibitors on *C. gigas* fecundation. Dose-response fertilization assays in the presence of ACE inhibitors. The fecundation efficiency is given as the percentage of segmented eggs relative to controls two hours after oocytes and sperm were mixed. The inhibitors (captopril, lisinorpil and fosinoprilat) and concentrations used (x axis) are indicated. ***: p<0.0001, two-way ANOVA, effect of concentration for each inhibitor.

All the inhibitors induced a dose-dependent decrease in the fecundation rate (p<0.0001). The IC_50_ values are ca. 5.10^−6^, 5.10^−5^ and 5.10^−3^ mol.L^−1^ for lisinopril, fosinoprilat and captopril, respectively, indicating that the enzymatic activity of *Cg*ACE is crucial for fertility in *C. gigas* (Figure 6).

## Discussion

This study presents the characterization of a functional angiotensin-converting enzyme orthologue in the oyster *Crassostrea gigas*, termed *Cg*ACE. This work describes the cloning, expression, structural modelling, function and biological characterization of the enzyme. To our knowledge, *Cg*ACE is not only the first ACE orthologue characterized in molluscs, but also the first to be given a biological function outside Vertebrates and Ecdysozoans. The oyster enzyme characterization may help to answer numerous questions that remain unclear, such as functional issues along with ACE evolution.

The oyster ACE orthologue, *Cg*ACE, resembles the human germinal isoform. As revealed by western blotting, the molecular weight of the mature protein is consistent with the *in silico* predictions. Futhermore, it also correlates the actual size of single active site ACE-like enzymes found from Insects up to Mammals. *Cg*ACE bears a unique active site and retains all the residues supporting both the catalytic activity and the interactions with inhibitors such as captopril and lisinopril (see results). These observations are in line with the finding that *Cg*ACE exhibits an ACE-like activity which is sensitive to ACE inhibitors. Accordingly, recombinant *Cg*ACE hydrolyses synthetic Angiotensin I and FRK peptides far less efficiently than the recombinant HswtACE, even when taking the number of active sites into account. However, the homology modelling clearly indicates that *Cg*ACE displays a wider catalytic channel than the human tACE, suggesting more permissive biochemical behaviour. Such a conformation of the active site, where substrates/inhibitors would ‘loosely’ fit, also probably explains why the classical ACE inhibitors seem less potent on recombinant oyster ACE than on human ACE. Indeed, despite captopril, lisinopril and fosinoprilat efficiently prevent *Cg*ACE activity on FRK peptide at low concentrations (i.e. 10^−5^ mol.L^−1^), such an inhibition is less potent than for the human recombinant protein extract. Similarly, in the presence of 10^−6^ mol.L^−1^ captopril, angiotensin II was detected by mass spectrometry after angiotensin I hydrolysis by recombinant *Cg*ACE, but not by recombinant HswtACE (data not shown), indicating that the inhibition of *Cg*ACE is somehow incomplete. Accordingly, and because three different peptides are *in vitro* substrates for *Cg*ACE, it is not excluded that the enzyme may hydrolyse a wide range of substrates. Nevertheless, one should keep in mind that the biochemical data herein originates from unpurified recombinant oyster ACE expressed in mammalian cells. Such methodology was widely demonstrated to be robust enough to assess the protease activity of recombinant ACE-like enzymes [4], [30], [31], [48], but may not adequately reflect the precise and specific biochemistry features of the native *Cg*ACE, which were not investigated further. Such an explanation might also hold true for the N- or C-domain specificity of inhibitors. Indeed, both captopril and lisinopril are able to inhibit *Cg*ACE activity on the FRK substrate. The ‘N-domain-like’ influence of chloride ion concentration on HHL hydrolysis suggests that *Cg*ACE may resemble the N-domain of mammalian sACE, and this despite showing a higher similarity with tACE. This finding is in line with what was observed for *Tt*ACE in the leech, also belonging to Lophotrochozoans. In contrast with the chloride concentration influence, phylogenetic analyses do not indicate that the oyster and leech ACE ortholgues are closely related to one another. Indeed, drosophila ACEs intercalate between them, and *Cg*ACE appears close to the bacterial *Xc*ACE, whereas *Tt*ACE clusters with vertebrate isoforms. Because both leeches and oysters belong to the Lophotrochozoa, such an observation might reflect complex evolutionary relationships between annelids and molluscs, and may also involve the specific parasitic trait of leeches, as discussed elsewhere [30]. Besides, the finding of a unique catalytic region within *Cg*ACE, and more generally within all lophotrochozoan ACE-like enzymes described to date [30], seems consistent with the late occurrence of genome duplication during the course of animal evolution. Indeed, this duplication occurred far after the Protostomes-Deuterostomes divergence and is widely admitted to explain the presence of two active site ACE-coding sequences in vertebrate genomes, such as the human *ace-1* gene [3]. *In silico* hydrophobicity predictions indicate the presence of a transmembrane domain in the carboxy-terminal region of the mature oyster ACE. Furthermore, membrane fractions of cells expressing *Cg*ACE exhibit ACE activity. In addition, though such information should be carefully interpreted, cells expressing the transmembrane human wild-type somatic ACE, and cells expressing *Cg*ACE, display similar ACE immunoreactivity suggesting similar recombinant protein localizations. Altogether, these results indicate that, very surprisingly, *Cg*ACE possesses a C-terminal transmembrane anchor. To our knowledge, *Cg*ACE is the first transmembrane ACE-like enzyme ever characterized outside of vertebrates and ecdysozoans. Besides, ACE activity is also found in the soluble fractions and culture media of transfected cells as well. Moreover, *Cg*ACE and tACE display high similarity within the stalk region lying upstream from the transmembrane domain (see Supplementary Figure S1). Consequently the oyster enzyme, like its human counterpart, putatively undergoes post-translational shedding that would release soluble recombinant *Cg*ACE from the CHO cells membrane. However, because the presence of an ACE-secretase in oysters is not demonstrated, the biological significance of such phenomenon remains elusive. Nevertheless, the oyster ACE displays a C-terminal transmembrane anchor, indicating that the appearance of this feature within ACE could lye before the Bilaterians division, and thus be more ancient than what was previously hypothesized [31].

Oysters are seawater organisms that lack a closed circulatory system and therefore do not display blood pressure regulation issues like Mammals do. Regardless of this point, the presence of ACE in oysters is consistent with the presence of ACE orthologues throughout the animal kingdom [30], [31]. However, the biological function(s) of such orthologues has never been addressed outside Vertebrates and Ecdysozoans, despite being of interest for the understanding of the evolution of enzyme/substrates systems. The absence of a model species with highly developed molecular and functional tools in Lophotrochozoans, and the great diversity of organisms within this group are certainly participating in such a poor understanding. However, RT-qPCR and *in situ* hybridization experiments reveal that, within somatic tissues, the highest *Cg*ACE expression levels are observed in the oyster's digestive tract, i.e. labial palps, gills and digestive gland. This expression pattern likely reflects a digestive and unspecialized role of the enzyme, like already speculated for ACE orthologues in all the other metazoan organisms in which ACE expression was examined [31], [49]. Consistently, the greatest amounts of *Cg*ACE mRNA during oyster development are observed just before the metamorphosis when the animals undergo morpho-physiological changes implicating a degradation of many cell proteins. However, very interestingly, the main site of *Cg*ACE mRNA and protein synthesis appears to be the germinal compartment within the gonadal area, but only in male oysters. *Cg*ACE mRNA levels dramatically increase in the male germinal tract along with spermatogenesis. These mRNAs are likely synthesized in the germinal cells because spermatogonia, spermatocytes I and spermatocytes II display strong labelling. In contrast, spermatids and mature spermatozoids exhibit little if any signal, in line with their low and selective transcriptional activity (for review see [50], [51]). Because the number of germinal cells increases during sexual maturation, the increasing amounts of *Cg*ACE transcripts likely originate in the growing number of germinal cells expressing stable levels of *Cg*ACE mRNA. Nevertheless, because the whole gonadal area was assayed in RT-qPCR, an increase in the individual *Cg*ACE transcription within each germinal cell cannot be excluded. Besides, the transfer of mature transmembrane enzymes has, to the best of our awareness, never been demonstrated. Therefore, the ‘Sertoli-like’ intra-tubular somatic cells are unlikely to synthesize *Cg*ACE, though (i) *in situ* results cannot strictly rule out such expression, and (ii) a feeder role is presumed for those cells. Immunolocalisation strongly correlates with this mRNA expression pattern, since the *Cg*ACE protein seems to be present within the gonadic tubules from early (stage I) to late stages (spermatozoids) of male gametogenesis. Moreover, a strong specific ACE activity is found in oyster sperm protein extracts, indicating that the mature enzyme accumulates into spermatozoids, but not into the surrounding fluid, in contrast with what was observed in the crayfish [19]. Interestingly, non negligible levels of ACE activity were also found in oocyte protein extracts, whereas both oocytes and the female gonadal area hardly transcribe *Cg*ACE. Such activity could come from solubilised digestive ACE which would then accumulate in the developing ovary and help generate peptides for vitellogenesis, mimicking the situation already observed in the mosquito [25].

A finding of interest lies in the assessment of the biological function of *Cg*ACE. Since ACE inhibitors are active on *Cg*ACE, they were used as functional tools in oyster fecundation assays, resulting in a dramatic and dose-dependent decrease in the fertilization rate. Most of this effect should be attributed to the inhibition of sperm ACE activity. Indeed, the very little ACE activity within mature oocytes is unlikely to be significant in this context. Because captopril, lisinopril and fosinoprilat are efficient on both recombinant *Cg*ACE and oyster sperm extracts, our results indicate that the peptidase activity of *Cg*ACE is crucial for fertilization in oysters. Even though high inhibitor concentrations could not totally prevent eggs fertilization (∼10 to 20% of the oocytes still undergo cleavage whatever the concentrations used), their effect is highly potent. Captopril displays a higher IC_50_ but its effect is harder to make clear because of the presence of thiol groups which could (i) compromise the molecular stability in sea water and (ii) become toxic for embryo cleavage at high concentrations. Furthermore, because human ACE inhibitors do not completely block *Cg*ACE activity in all the reaction conditions we assayed, one could argue that a fully potent specific *Cg*ACE inhibitor could completely block oyster fecundation. Besides, the presence of another oyster ACE-like orthologue cannot be excluded, which would be little if at all sensitive to the inhibitors we used. Such concerns could be addressed when the oyster genome becomes available. Alternatively, or in addition, other proteases than *Cg*ACE, that were not investigated, could also exist and exhibit similar functions, such as mouse ACR and PRSS21 serine protease orthologues [52]. The latter explanation seems more straightforward because such a basic and required process as the fecundation ability is likely to be rescued by compensatory mechanisms. Accordingly, expressed sequence tags presenting strong similarities with these two proteins exist within oyster libraries (data not shown). Nonetheless, IC_50_ values for lisinopril and captopril show evidence of the critical importance of *Cg*ACE, in a similar fashion to that of the human germinal ACE. Inhibition of the protease activity strongly suggests that *Cg*ACE would be expressed at the spermatozoid surface, and could degrade proteins of the chorionic membrane in order to help the male pronucleus penetrate the oocyte cytoplasm. Nevertheless, caution should be taken regarding this interpretation, because seawater chloride concentrations (c.a. 0,5 mol.L^−1^) are not optimal for recombinant *Cg*ACE activity. This may reflect the high physiological tolerance to freshwater of *C. gigas*. Otherwise, *Cg*ACE could exert additional physiological roles within the oyster extracellular compartment which we did not examine. Characterization of endogenous *Cg*ACE substrate(s) would clearly be of great help. However, many additional experiments are required in order to gain insights into these very interesting issues that lie beyond the focus of the present study.

Taken together, our results show that oysters express a functional ACE orthologue bearing a unique active site, *Cg*ACE. Interestingly, *Cg*ACE displays a transmembrane anchor, accumulates in spermatozoids, and is critical for fecundation through its peptidase activity. To date, *Cg*ACE is the only ACE orthologue to be assigned a biological function outside Vertebrates and Ecdysozoans. These findings not only help a better understanding of enzyme evolution, but also bring insights into the reproduction of the most important aquaculture resource worldwide.

## Supporting Information

Figure S1 **Multiple sequence alignment between** ***Cg*** **ACE and other ACE-like proteins widespread the animal kingdom.** The alignement was generated using the BLOSUM62 matrix; tACE, human tACE (GI: 23238214); Ndom, human sACE N-domain (GI: 113045 residues 1–612); ACE2, human ACE2 (GI: 42543475); AnCE, *Drosophila melanogaster* ACE (GI: 10728771); ACEr, *Drosophila melanogaster* ACE-related (GI: 17137262); ACN-1, *Caenorhabditis elegans* non-peptidase ACE (GI: 71985293); TtACE, leech *Theromyzon tessulatum* ACE (GI: 45272589); XcACE, *Xanthomonas axonopodis pv.citri* ACE (GI: 21241971); LdDCP, *Leishmania donovani* dicarboxypeptidase (GI: 56130986); CgACE: *Crassostrea gigas* ACE (Genbank accession number: JN382542). The similar (grey) and identical (black) residues between 80% of the sequences are shaded; the signal peptide cleavage site (↑), the gluzincin residues (A) and the putative transmembrane anchor (*) of *Cg*ACE are indicated below the alignment.
(DOC)

Figure S2 **MS/MS spectra of synthetic angiotensin II (A) and of the 1046 m/z peptide from hydrolysates of angiotensin I by** ***Cg*** **ACE (B).**
(DOC)
