# Supplementary material for: A Crucial Role in Fertility for the Oyster Angiotensin-Converting Enzyme Orthologue CgACE
Source: PLoS One. 2011 Dec 9;6(12):e27833. doi: 10.1371/journal.pone.0027833 (PMC3235092; doi:10.1371/journal.pone.0027833)
Supplement: Figure S1 — Multiple sequence alignment between Cg ACE and other ACE-like proteins widespread the animal kingdom. The alignement was generated using the BLOSUM62 matrix; tACE, human tACE (GI: 23238214); Ndom, human sACE N-domain (GI: 113045 residues 1–612); ACE2, human ACE2 (GI: 42543475); AnCE, Drosophila melanogaster ACE (GI: 10728771); ACEr, Drosophila melanogaster ACE-related (GI: 17137262); ACN-1, Caenorhabditis elegans non-peptidase ACE (GI: 71985293); TtACE, leech Theromyzon tessulatum ACE (GI: 45272589); XcACE, Xanthomonas axonopodis pv.citri ACE (GI: 21241971); LdDCP, Leishmania donovani dicarboxypeptidase (GI: 56130986); CgACE: Crassostrea gigas ACE (Genbank accession number: JN382542). The similar (grey) and identical (black) residues between 80% of the sequences are shaded; the signal peptide cleavage site (↑), the gluzincin residues (A) and the putative transmembrane anchor (*) of CgACE are indicated below the alignment. (DOC) [file pone.0027833.s001.doc]

10 20 30 40 50 60 70 80 90 100

....|....|....|....|....|....|....|....|....|....|....|....|....|....|....|....|....|....|....|....|

**Ndom**  **MGAASGRRGPGLLLPLPLLLLLPPQPALALDPGLQPGNFSADEAGAQLFAQSYNSSAEQVLFQSVAASWAHDTNITAENARRQEEAALLSQEFAEAWGQK**

**Cdom**  **-------------------------------------DLVTDEAEASKFVEEYDRTSQVVWNEYAEANWNYNTNITTETSKILLQKNMQIANHTLKYGTQ**

**TtACE** **-------------MNLINFSYLNLLFGAGLFSVLESATILNTESDAKKWLTTYNDEAGKYIYDATEAEWNYNTNLTDHNLGISIKKSNDLATFTEQKAIE**

**ACEr**  **-------MGACNITVLLLVIMLWLPHGLSMG-----NSCSASVLEARRFFELENEQLRRRFHEEFLSGYNYNTNVTEANRQAMIEVYARNAELNKRLAQQ**

**AnCE**  **----------------MRLFLLALLATLAVT-----QALVKEEIQAKEYLENLNKELAKRTNVETEAAWAYGSNITDENEKKKNEISAELAKFMKEVASD**

**ACE2**  **----------------MSSSSWLLLSLVAVT-----AAQSTIEEQAKTFLDKFNHEAEDLFYQSSLASWNYNTNITEENVQNMNNAGDKWSAFLKEQSTL**

**CgACE** **--MKALWRLSVLCVLCTCVIARHRHDSQQRLRQVLQQRRQRIDTEVSEWPTTKNEELRRWLEKISFASWNYETDLSEENAEKLNKVNIDFSQWKSTCVKE**

**XcACE** **---MNPRLLLLALAVATGTLSLSACRRDAAPPDKPVATKTAPAESADQFVARISAEYKAAYPETTAAQWLSSTYINGDSQLLAAKANERSLAQLDRWIEQ**

**LdDCP** **-------MSANPLLQQSTLQYQYPPFDRIAVEHYAPAFEQGMAEQMAEIEVIKSNPDAPTLENTVVALERSGAQLKRARLVFQNLCSAHTNPEMQSLEQA**

**ACN-1** **-------------------------------------------MKFHILLLLLVGACLPVFTQEIKPKPELLPADEAPKDPEAVFSEGEPFELTDALDTP**

↑

110 120 130 140 150 160 170 180 190 200

....|....|....|....|....|....|....|....|....|....|....|....|....|....|....|....|....|....|....|....|

**Ndom**  **AKELYEPIWQNFTDPQLRRIIGAVRTLGSANLPLAKRQQYNALLSNMSRIYSTAKVC-LPNK-TATCWSLDPDLTNILASSRSYAMLLFAWEGWHNAAGI**

**Cdom**  **ARKFDVNQLQNTT---IKRIIKKVQDLERAALPAQELEEYNKILLDMETTYSVATVC-HPN---GSCLQLEPDLTNVMATSRKYEDLLWAWEGWRDKAGR**

**TtACE** **ANKKFV--WKNFTDPLLKREFSKITDIGTASLSDEDFQKMSGLNSDLTKIYSTAKVCNKPNDPSGKCYPLDPDLSDIISKSNDLEELTWAWKGWRDASGK**

**ACEr**  **IKSSDYVQSEDAD---IRRQAEHLSKLGASALNADDYLALQNAISSMQTNYATATVCSYTNR-SDCSLTLEPHIQERLSHSRDPAELAWYWREWHDKSGT**

**AnCE**  **TTKFQWRSYQSED---LKRQFKALTKLGYAALPEDDYAELLDTLSAMESNFAKVKVCDYKDS-TKCDLALDPEIEEVISKSRDHEELAYYWREFYDKAGT**

**ACE2**  **AQMYPLQEIQNLT---VKLQLQALQQNGSSVLSEDKSKRLNTILNTMSTIYSTGKVCNPDNP--QECLLLEPGLNEIMANSLDYNERLWAWESWRSEVGK**

**CgACE** **ASEKLKFPGLSED----ARHQLRILSYDIDPSNPEQLRKVNDIQTKLEQIYGRGTVEYNGTT-----LQLEPGITNLFESSRDPKVLSELWVKWRDATGR**

**XcACE** **SKQYAGTPMSADS--ARALQLLKLMSALPAPRDPAKLAELTRIAAKMEGDYGAGSYCVGEGE--QRRCRQLGELEQVLASSRDYNEQLDAWQGWHS-TAQ**

**LdDCP** **YAPKFAVHTDKIYLDGALYHRIKAVYDARASLAGEDLR----LVEHYEREFRKAGAALHDSD-KEKLKQVNERLATLESDFAKKVMGTRKTASLVVDDVA**

**ACN-1** **KNGSVPVPEPEPKPEPEPEPEPKPEPEPSPTPEPEPAIKFDNIESEDYGDVAETAASTQPDE-------LNTEVIEQLVDT-------------------**

210 220 230 240 250 260 270 280 290 300

....|....|....|....|....|....|....|....|....|....|....|....|....|....|....|....|....|....|....|....|

**Ndom**  **PLKPLYEDFTALSNEAYKQDGFTDTGAYWRS--WYNSP----------TFEDDLEHLYQQLEPLYLNLHAFVRRALHRRYG----DRYINLRGPIPAHLL**

**Cdom**  **AILQFYPKYVELINQAARLNGYVDAGDSWRS--MYETP----------SLEQDLERLFQELQPLYLNLHAYVRRALHRHYG----AQHINLEGPIPAHLL**

**TtACE** **HMPDKYDEFVQLLNKAANINGYEDNGDYWRS--WYESP----------TFRKDCEDLWQEIKPFYEQLHAYVRRKLQKKYP----QIAFPKEGHIPAHLL**

**ACEr**  **PMRQNFAEYVRLTRKASQLNGHRSYADYWVQ--FYEDP----------DFERQLDATFKQLLPFYRQLHGYVRFRLRQHYG----PDVMPAEGNIPISLL**

**AnCE**  **AVRSQFERYVELNTKAAKLNNFTSGAEAWLD--EYEDD----------TFEQQLEDIFADIRPLYQQIHGYVRFRLRKHYG----DAVVSETGPIPMHLL**

**ACE2**  **QLRPLYEEYVVLKNEMARANHYEDYGDYWRG--DYEVNGVDGYDYSRGQLIEDVEHTFEEIKPLYEHLHAYVRAKLMNAY-----PSYISPIGCLPAHLL**

**CgACE** **KMANLYTEFVELQNTGALEHGFEDLGDAWRQREFFDTP----------GLVQIVEDLWQELRPMYVQLHSYVRRKLETYYAKNHPDFDFPKDGSIPAHLL**

**XcACE** **PMRKDYQRFVELANEGARGLGFADVGVLWRSG--YDMP--------PAQLASETDRLWEQVKPLYAQLQCYARGKLDTQYGK---DKGEVAGGLLPAHLM**

**LdDCP** **ELEGLSEDEIATAQKEAESLGHPGKYALIIVN-TTQQP-----------LLASLRSRETRRRLFEASVQRAGRGDENDTSA---IIVEIAQLRLRKARLL**

**ACN-1** **-----------FLNTGSIASNKTNKGPVFANP--------------------------VAQALVNLTLNHFSSTTSHSSTG--------SAIPMKSIRLL**

310 320 330 340 350 360 370 380 390 400

....|....|....|....|....|....|....|....|....|....|....|....|....|....|....|....|....|....|....|....|

**Ndom**  **GDMWAQSWENIYDMVVPFPDKPNL-DVTSTMLQQGWN------------------------------ATHMFRVAEEFFTSLELSPMPPEFWEGSMLEKP**

**Cdom**  **GNMWAQTWSNIYDLVVPFPSAPSM-DTTEAMLKQGWT------------------------------PRRMFKEADDFFTSLGLLPVPPEFWNKSMLEKP**

**TtACE** **GNMWAQSWENIEYLLRPAPDLPSM-DITEELVKQNYT------------------------------ALKLFQLSDTFFKSLGLIQMPQPFWEKSMIEKP**

**ACEr**  **GNMWGQSWNELLDLFTPYPEKPFV-DVKAEMEKQGYT------------------------------VQKLFELGDQFFQSLGMRALPPSFWNLSVLTRP**

**AnCE**  **GNMWAQQWSEIADIVSPFPEKPLV-DVSAEMEKQGYT------------------------------PLKMFQMGDDFFTSMNLTKLPQDFWDKSIIEKP**

**ACE2**  **GDMWGRFWTNLYSLTVPFGQKPNI-DVTDAMVDQAWD------------------------------AQRIFKEAEKFFVSVGLPNMTQGFWENSMLTDP**

**CgACE** **GNMWAQEWNNIFDIVQPYPEVKEP-DTNRALKEKGYN------------------------------VGSMFRRAEEFYTSLGLYKMVPDFWRRSMLVRP**

**XcACE** **GNMWQQDWSNLWDLLQPYPGAGDL-DITSALEKQYQGNLTAVLARNASGDGGAAARFNAEREAQLRTAKQMTERAQDFYTSLGMPKLPDTYWQRSQFIKP**

**LdDCP** **GKKSFSEWQLQNQMADPASAEALLRDMGDAAASKVKR-----------------------------EAADIKQMIREEGGDFELAPWDWKYYAERVRKQR**

**ACN-1** **LDGTEKELHSLCSEEIPKTRQPGD-------------------------------------------------------GGNGLPSEDRVAFPGGECVNG**

410 420 430 440 450 460 470 480 490 500

....|....|....|....|....|....|....|....|....|....|....|....|....|....|....|....|....|....|....|....|

**Ndom**  **ADG-REVVCHASAWDF--------YNRKDFRIKQCTRVTMDQLSTVHHEMGHIQYYLQYKDLPVSLRRGANPGFHEAIGDVLALSVSTPEHLHKIGLLD-**

**Cdom**  **TDG-REVVCHASAWDF--------YNGKDFRIKQCTTVNLEDLVVAHHEMGHIQYFMQYKDLPVALREGANPGFHEAIGDVLALSVSTPKHLHSLNLLS-**

**TtACE** **AD--RDVVCHASAWDF--------YNRKDFRIKQCTVVDMHWFMTTHHEMGHIEYYLHYKDQPISFRSGANPGFHEAIADIASLSVATPEYMQSVSLLP-**

**ACEr**  **DD--RHVVCHASAWDF--------YQDSDVRIKMCTEVDSHYFYVVHHELGHIQYYLQYEQQPAVYRGAPNPGFHEAVGDVIALSVMSAKHLKAIGLIE-**

**AnCE**  **TDG-RDLVCHASAWDF--------YLTDDVRIKQCTRVTQDQLFTVHHELGHIQYFLQYQHQPFVYRTGANPGFHEAVGDVLSLSVSTPKHLEKIGLLK-**

**ACE2**  **GNV-QKAVCHPTAWDL--------GKG-DFRILMCTKVTMDDFLTAHHEMGHIQYDMAYAAQPFLLRNGANEGFHEAVGEIMSLSAATPKHLKSIGLLSP**

**CgACE** **TEKDREVQCHASAFDF--------YNNDTFRIKMCTEVNMNYFQTIHHEMGHIEYFMAYRHRPTVYGKGANGGFHEAIGDTIALSVRSRKHLQTLGLLED**

**XcACE** **LDR--DVVCHASAWDMNMGGEASQNIGADVRTKMCIKPTEEDFTTIYHELGHIYYDLAYNPLPPLFQNGANDGFHEAIGDTIVLAMT-PKYLQSIGMVGE**

**LdDCP** **YDLDENETKPYFELNNVLERG---VFYTAEKLYGVTMQRRTDLPVYHPDVMSFEMFDCTGESLAIFCLDPYARASKRGGAWMTFYVRQSSLLGQKPVVYN**

**ACN-1** **QECLLDSHCNG------------------------------TICVCNDGLYTLEIGNTFN---CVPGNPADSGFGDGKGGLV------------IGLFNN**

AAAAAA A

510 520 530 540 550 560 570 580 590 600

....|....|....|....|....|....|....|....|....|....|....|....|....|....|....|....|....|....|....|....|

**Ndom**  **RVTNDTE---SDINYLLKMALEKIAFLPFGY----LVDQWRWGVFSGRTPP------SRYNFDWWYLRTKYQGICPPVTRNETHFDAGAKFHVPNVTPYI**

**Cdom**  **SEGGSDE---HDINFLMKMALDKIAFIPFSY----LVDQWRWRVFDGSITK------ENYNQEWWSLRLKYQGLCPPVPRTQGDFDPGAKFHIPSSVPYI**

**TtACE** **NFTDDPN---GDLNFLMNQALTKVAFLPFGY----LIDQWRWDVFSGDTPR------PKYNSKWWHNRCKYQGVYPPVIRSEQDFDAGSKFHVPNNTPYI**

**ACEr**  **NGRLDEK---SRINQLFKQALSKIVFLPFGY----AVDKYRYAVFRNELDE------SQWNCGFWQMRSEFGGVEPPVFRTEKDFDPPAKYHIDADVEYL**

**AnCE**  **DYVRDDE---ARINQLFLTALDKIVFLPFAF----TMDKYRWSLFRGEVDK------ANWNCAFWKLRDEYSGIEPPVVRSEKDFDAPAKYHISADVEYL**

**ACE2**  **DFQEDNE---TEINFLLKQALTIVGTLPFTY----MLEKWRWMVFKGEIPK------DQWMKKWWEMKREIVGVVEPVPHDETYCDPASLFHVSNDYSFI**

**CgACE** **ANVSEEEKKKSDINFLLNQALLKLAFLPFGY----LVDKWRWRVFSGEITP------DKYNEEWWKMRLEYQGVTSPVPRTNSDFDPGAKFHVAANSQYI**

**XcACE** **QQTGRES----LINSQMRMALSKVAFLPFGL----MIDRWRWGVFDGSITP------EHYNQAWWELKAKYQGVAPVSARGEEFFDPGAKYHVPGNTPYT**

**LdDCP** **VLNVVKP--AEGRPTLLSRSDVTTLFHEFGHGLHGMLSNLKYSTLSGTSVARDFLEFPSQINEHWAMYDAVLKNYAIHYETKEPIPQALVDRMKAAETYG**

**ACN-1** **EVTTPEP----------------------------------------SAEP------------------------EPTAKTTTKMPPRVRAATSPFSLYL**

*******

610 620 630 640 650 660 670 680 690 700

....|....|....|....|....|....|....|....|....|....|....|....|....|....|....|....|....|....|....|....|

**Ndom**  **RYFVSFVLQFQFHEALCKEAGYEGP------LHQCDIYRSTKAGAKLRKVLQAGSSRPWQEVLKDMVGLDALDAQPLLKYFQPVTQWLQE----------**

**Cdom**  **RYFVSFIIQFQFHEALCQAAGHTGP------LHKCDIYQSKEAGQRLATAMKLGFSRPWPEAMQLITGQPNMSASAMLSYFKPLLDWLRTENELHGEKLG**

**TtACE** **RYFVAHIIQFQFHEALCKAANNSRP------LHRCNIANSKEAGKKLAELMKSGSSIPWPKVLENLTGSEKMSAKSLMAYYKPLIDWPEKRKPRAENWMG**

**ACEr**  **RYFAAHIFQFQFHKALCRKAGQYAPNNSRLTLDNCDIFGSKAAGRSLSQFLSKGNSRHWKEVLEEFTGETEMDPAALLEYFEPLYQWLKQENSRLGVPLG**

**AnCE**  **RYLVSFIIQFQFYKSACIKAGQYDPDNVELPLDNCDIYGSAAAGAAFHNMLSMGASKPWPDALEAFNGERIMSGKAIAEYFEPLRVWLEAENIKNNVHIG**

**ACE2**  **RYYTRTLYQFQFQEALCQAAKHEGP------LHKCDISNSTEAGQKLFNMLRLGKSEPWTLALENVVGAKNMNVRPLLNYFEPLFTWLKDQN--KNSFVG**

**CgACE** **IYFASFLLQFQFYESMCQASGHTGP------LYTCDFYRSKEAGGKLLNMLKLGGSKHWKEALKQMTGDTNIRTEPFKKYFQPLIDFLMNEN---KGDVG**

**XcACE** **RYFLAHILQFQFYKGLCQAAGHQGP------LYECSFYGNKDAGQKFWSMLQRGSSQPWQTTLKELTGNDKLDAGPMLEYFAPMSDWLKQQN--QGQMCG**

**LdDCP** **AGFHTIEVVKAAYLDLCWHLVAEETAVLP--PAQMEEAAMKSFGVGMTEVPPRYHSGYFMHTFSGGYASNYYVYQWARVLDCDGFEWFLENGGLTRENGD**

**ACN-1** **TVLLIIYFAL------------------------------------------------------------------------------------------**

*************

710 720 730 740 750 760 770 780

....|....|....|....|....|....|....|....|....|....|....|....|....|....|....|....|....|.

**Ndom**  **--------------------------------------------------------------------------------------**

**Cdom**  **WPQYNWTPNSARSEGPLPDSGRVSFLGLDLDAQQARVGQWLLLFLGIALLVATLGLSQRLFSIRHRSLHRHSHGPQFGSEVELRHS**

**TtACE** **GKMSSWIV------------------------------------------------------------------------------**

**ACEr**  **WGPTDKIPSDCCGTFST---------------------------------------------------------------------**

**AnCE**  **WTTSNKCVSS----------------------------------------------------------------------------**

**ACE2**  **WSTDWSPYAD----------------------------------------------------------------------------**

**CgACE** **WAKAGINWERA---------------------------------------------------------------------------**

**XcACE** **WQATAAAPVTTKTAPAAAR-------------------------------------------------------------------**

**LdDCP** **HLRACVLSVGNSVDANVAYEKFAGRKANMKAFLRINGLLDE---------------------------------------------**

**ACN-1** **--------------------------------------------------------------------------------------**
