# Supplementary figures and images for: A Crucial Role in Fertility for the Oyster Angiotensin-Converting Enzyme Orthologue CgACE
Source: PLoS One. 2011 Dec 9;6(12):e27833. doi: 10.1371/journal.pone.0027833 (PMC3235092; doi:10.1371/journal.pone.0027833)

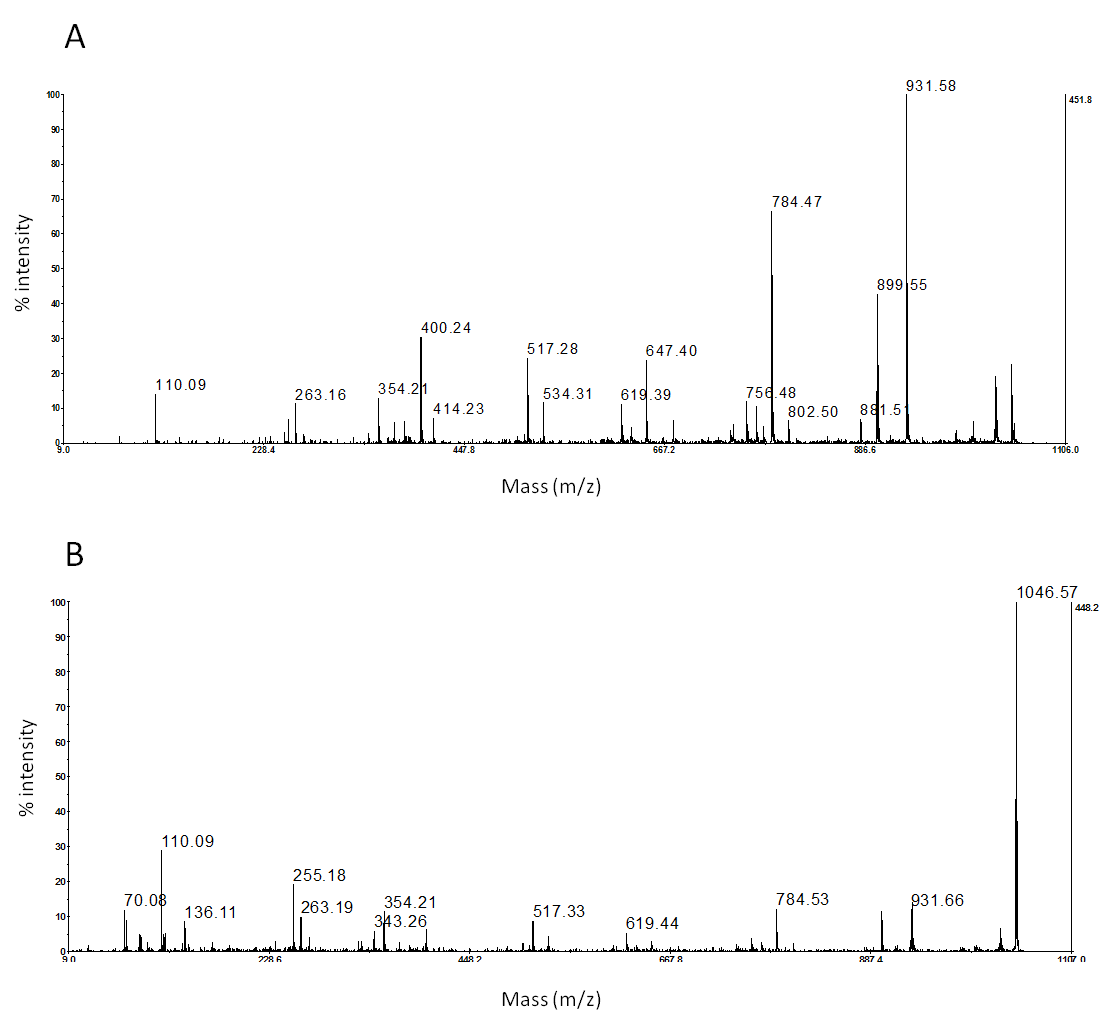

Supplement: Figure S2 — MS/MS spectra of synthetic angiotensin II (A) and of the 1046 m/z peptide from hydrolysates of angiotensin I by Cg ACE (B). (DOC) [file pone.0027833.s002.doc]
